# Assessment of symptoms in myalgic encephalomyelitis/chronic fatigue syndrome: a comparative study of existing scales

**DOI:** 10.3389/fneur.2025.1618272

**Published:** 2025-11-18

**Authors:** Jing Lu, Weibo Sun, Shulin Li, Yuanyuan Qu, Tingting Liu, Shuhao Guo, Chuwen Feng, Tiansong Yang

**Affiliations:** 1 Heilongjiang University of Chinese Medicine, Harbin, China; 2 Department of Breast Surgery, Harbin Medical University Cancer Hospital, Harbin, China; 3 Harbin Medical University, Harbin, China; 4 Rehabilitation Department II, The First Affiliated Hospital of Heilongjiang University of Chinese Medicine, Harbin, Heilongjiang, China; 5 Heilongjiang Province Key Laboratory of Traditional Chinese Medicine Information, Harbin, Heilongjiang, China

**Keywords:** myalgic encephalomyelitis/chronic fatigue syndrome, assessment scales, symptom assessment, fatigue, clinical instruments

## Abstract

Myalgic encephalomyelitis/chronic fatigue syndrome (ME/CFS) is a multifaceted disorder characterized by persistent fatigue, post-exertional malaise (PEM), cognitive dysfunction, sleep disturbance, pain, psychological distress, orthostatic intolerance, and impaired multidimensional health status and functioning. In the absence of reliable biomarkers, standardized symptom assessment is essential for accurate diagnosis and comparability across studies. This narrative literature review synthesized studies identified through PubMed and Web of Science up to June 2024, covering assessment instruments across major ME/CFS symptom domains. Tools were evaluated for their psychometric validity, clinical applicability, and key limitations. Overall, existing scales demonstrate acceptable reliability but vary in sensitivity and disease specificity. Harmonized, multidimensional, and digitally or objectively validated measures are needed to improve diagnostic precision, longitudinal monitoring, and clinical translation in ME/CFS.

## 1 Introduction

Myalgic encephalomyelitis/chronic fatigue syndrome (ME/CFS) is a chronic, disabling disorder of unknown etiology, with no established biomarkers or curative treatment (1). Diagnostic frameworks have evolved from the broad Fukuda criteria (1994) through the Canadian Consensus Criteria (2003), to the IOM/NAM framework (2015), which identified post-exertional malaise (PEM) as the hallmark feature, alongside sleep, pain, cognitive, and autonomic/immune disturbances (1–3). More recently, the EUROMENE guidelines (2021) emphasized harmonization of diagnostic and research standards (4).

Recent U.S. survey data estimated a prevalence of 1.3% among adults, with higher rates in women than men (5). A meta-analysis of long COVID cohorts reported that 8.4% fulfilled ME/CFS criteria, underscoring the role of post-viral syndromes in disease burden (6).

In the absence of objective biomarkers, validated symptom assessment scales remain essential for clinical evaluation, disease monitoring, and research comparability (7). This review synthesizes established and emerging instruments across key symptom domains, critically appraises their validity and applicability, and provides comparative tables and practical recommendations to identify suitable tools, highlight limitations, and outline priorities for future research in ME/CFS.

## 2 Methods

This narrative literature review was conducted by searching PubMed and Web of Science for studies published up to June 2024 using the following search terms: (“myalgic encephalomyelitis” OR “chronic fatigue syndrome” OR ME/CFS) AND (“assessment” OR “scale” OR “questionnaire”) AND (“fatigue” OR “post-exertional malaise” OR “cognition” OR “sleep” OR “pain” OR “psychological state” OR “orthostatic intolerance” OR “quality of life”). Studies were included if they described the development, validation, or clinical application of ME/CFS assessment tools. Non-peer-reviewed articles and studies without psychometric validation were excluded. Scales were selected based on their frequency of use in ME/CFS research, psychometric validation, and clinical applicability. Titles and abstracts were screened by two independent reviewers, and disagreements were resolved by discussion. Additional studies were identified through reference tracking of key articles to ensure comprehensive inclusion of relevant studies. As a narrative review, no formal risk-of-bias assessment was performed, but transparent reporting of the selection criteria was adhered to. For consistency, the term ME/CFS is used throughout this review to refer to studies employing various case definitions, including Fukuda 1994, CCC 2003, and IOM/NAM 2015.

## 3 Fatigue-related assessment scale

Fatigue is the core symptom of ME/CFS, related scales are widely applied in clinical studies, mostly assessing physical and mental fatigue. With continued refinement, these instruments now cover broader domains and are also used in other fatigue-associated conditions such as cancer (8), multiple sclerosis (9) and epilepsy (10).

### 3.1 Chalder fatigue questionnaire (CFQ)

The Chalder fatigue scale (CFQ or CFS or FS-14), developed by Butler and refined by Chalder in 1991 (11), is among the most widely used fatigue tools. It contains 14 items measuring physical and mental fatigue and can distinguish fatigued individuals from healthy controls and track fatigue changes in ME/CFS (12). However, Kirke argued that the FS-14 is inadequately captures changes in ME/CFS fatigue. His study found that a two-point reduction in score increases bias toward perceived symptom improvement, whereas a 10-point reduction enhances the accuracy of outcome interpretation (13). To address these limitations, a shortened version, the CFQ-11, was later developed by removing the items “easy start,” “clear thinking,” and “loss of interest,” reducing the total from 14 to 11 items. Items 1–7 assess physical fatigue, and 8–11 assess mental fatigue (14). Structural equation modeling confirmed that the CFQ-11 achieved a better three-factor structure than the FS-14 (15). Although the FS-14 remains widely used in ME/CFS research, the CFQ11 demonstrates improved structural validity and may provide more reliable fatigue assessments.

### 3.2 Fatigue assessment instrument (FAI)

The fatigue assessment instrument (FAI), developed by Schwartz and Jandorf (16), consists of 29 items assessing the past 2 weeks across four domains: fatigue severity, sensitivity, psychological impact, and the effect on rest or sleep. The scale was designed to measure both the quantitative and qualitative components of fatigue (17), qualitatively to determine whether fatigue is persistent and physiological or transient and physiological in healthy individuals, and quantitatively to evaluate the graded severity of fatigue, with higher total scores indicating more severe symptoms (16). Despite its application in ME/CFS research, psychometric validation remains limited, and further studies are needed to confirm its reliability and validity.

### 3.3 Fatigue severity scale (FSS)

The fatigue severity scale (FSS), developed by Krupp (18) for multiple sclerosis (MS) and systemic lupus erythematosus (SLE), is now widely applied in studies of ME/CFS (19) and cancer-related fatigue (20). It consists of nine items rated on a seven-point Likert scale ranging from 1 (strongly disagree) to 7 (strongly agree), with higher scores indicating greater fatigue severity (21). The FSS primarily reflects the impact of fatigue on daily life rather than the intensity of symptoms (22). Psychometric studies have demonstrated high validity, internal consistency, and test-retest reliability, supporting its use in follow-up assessments and treatment evaluation (23).

### 3.4 Fatigue impact scale (FIS)

The fatigue impact scale (FIS), developed by Fisk in 1994, is a multidimensional instrument designed to evaluate patients' fatigue during the previous month across three domains: cognitive, physical (somatic) and psychological functioning. It primarily reflects the impact of fatigue on patients' ability to perform daily and social activities (24). The FIS comprises 40 items, each rated on a five-point scale from 0 (no fatigue) to 4 (very severe fatigue), with higher scores indicating greater impairment in the respective domain (25). A 21-item modified version (MFIS), retaining the same domains and scoring method, was later adopted by the U.S. National Multiple Sclerosis Society for inclusion in its 1998 Clinical Practice Guidelines as a measure of fatigue impact on daily living (26). The MFIS has since been widely applied in ME/CFS research (19, 27).

### 3.5 Checklist individual strength (CIS)

The checklist individual strength (CIS), developed by the University Medical Centers of Amsterdam and Rotterdam in 1994, is a multidimensional instrument designed to evaluate self-perceived fatigue over the preceding 1–2 weeks (28). It has been widely applied in studies of ME/CFS (29), cancer (30) and multiple sclerosis (31), as well as in epidemiological studies of healthy working populations. It has also been validated in rheumatoid arthritis (RA) (32) and fibromyalgia (33). The scale comprises 20 items across four domains: fatigue severity, attention, motivation and activity level, each rated on a seven-point Likert scale, with higher scores indicating more severe physical and mental fatigue (34). Psychometric analyses have reported a Cronbach's α of ~0.90, demonstrating high internal consistency, test-retest reliability and sensitivity to change (35), and validated cut-offs for severe fatigue (36). Compared to other scales, the CIS places greater emphasis on cognitive aspects of fatigue in ME/CFS.

### 3.6 Visual analog scale for fatigue (VAS-F)

The visual analog scale for fatigue (VAS-F), developed by Lee et al. (37), was adapted from general visual analog scales (VAS) previously used to quantify subjective emotional or affective states, and designed as a rapid, quantitative measure of fatigue. It was demonstrated to be a valid and reliable unidimensional measure, and has since been applied in studies of inflammatory bowel disease (38), ME/CFS (27), MS (39), and primary Sjögren's syndrome (pSS) (40). The VAS-F consists of a 100 mm (10 cm) horizontal line, with one end labeled “no tiredness” and the other “maximum tiredness” or “extreme fatigue.” Participants mark a point along the line corresponding to their perceived fatigue intensity at that moment. The distance from the left anchor represents the fatigue score, with larger distances indicating greater fatigue severity. Although the VAS-F provides a simple and efficient measure of fatigue intensity, it cannot differentiate fatigue subcomponents (e.g., emotional, physical, or functional impact), and may be influenced by response bias and scoring variability, limiting its use for diagnostic purposes.

### 3.7 Other fatigue assessment scales

Additional instruments applied in ME/CFS research include the multidimensional fatigue inventory (MFI) (41), the PedsQL multidimensional fatigue scale (PedsQL-MFS) (42), the functional assessment of chronic illness therapy-fatigue scale (FACIT-F) (43), and the fatigue scale for motor and cognitive functioning (FSMC) (44). Each has distinct structural or dimensional characteristics but is used relatively infrequently, as their target populations or original design contexts differ from those of ME/CFS. The psychometric validity and reliability of these instruments in ME/CFS populations remain to be further established.

Table 1 summarizes the major fatigue-related scales, while detailed characteristics and supporting references for these additional scales are provided in Supplementary Table S1.

**Table 1:** Scales assessing fatigue commonly used in ME/CFS.

**Scale**	**Content of the assessment**	**Advantages**	**Limitations**
CFQ	14 items assessing physical and mental fatigue	Assesses physical and mental fatigue; widely used; distinguishes fatigued vs. healthy individuals; applicable across conditions	May overestimate improvement in ME/CFS; limited sensitivity to symptom worsening; no specificity
FAI	29 items, four domains (severity, sensitivity, psychological impact, rest/sleep impact)	Multidimensional assessment; evaluates both qualitative and quantitative fatigue features	Limited psychometric validation in ME/CFS
FSS	Nine items; measures fatigue impact on daily function	Short, reliable, widely used; emphasizes functional impact of fatigue	Emphasizes functional impact rather than symptom intensity; weak correlation with fatigue symptoms
FIS/MFIS	40/21 items, three domains (cognitive, physical, psychological)	Multidimensional; reliable; reflects cognitive, physical, and psychosocial fatigue	May not capture fatigue severity; limited discrimination from depression; possible ceiling effect
CIS	20 items, four domains (fatigue severity, attention, motivation, activity level)	Multidimensional; focuses on cognitive aspects; high internal consistency (α ≈ 0.90); sensitive to change	Limited sensitivity to long-term fatigue; requires self-report; limited objective validation
VAS-F	100 mm line from “no tiredness” to “extreme fatigue”	Quick; intuitive; quantitative measure of fatigue intensity	One-dimensional; cannot distinguish fatigue subcomponents; limited scoring precision
Other scales [MFI (45), PedsQL-MFS (46, 47)]	Multidimensional or population-specific fatigue measures	Broaden assessment scope; useful in pediatric, cancer, and neurological populations (45)	Less frequently validated in ME/CFS

Detailed characteristics and scoring information are provided in Supplementary Table S1.

## 4 Post-exertional malaise scales

Post-exertional malaise (PEM) is the hallmark symptom distinguishing ME/CFS from other fatigue-related conditions (4, 48), and its evaluation is critical for accurate diagnosis, disease monitoring, and research comparability. Several assessment tools have been developed to capture PEM features from both subjective and objective perspectives. Among them, the DePaul symptom questionnaire PEM subscale (DSQ-PEM) (49), the functional capacity scale (FUNCAP) (50), and the 2-day cardiopulmonary exercise test (2-day CPET) (51) are the most widely cited in current ME/CFS and post-COVID studies. Some multidomain symptom questionnaires or fatigue inventories, though not validated as dedicated PEM tools, still include PEM-related items and have been used exploratorily in clinical studies.

### 4.1 DePaul symptom questionnaire–PEM subscale (DSQ-PEM)

The DSQ-PEM is a subscale of the DePaul symptom questionnaire, developed by Leonard Jason's group at DePaul University to specifically assess PEM in ME/CFS cohorts (52). It typically includes five core items evaluating the frequency, severity, and duration of post-exertional symptoms such as fatigue relapse, cognitive decline, and flu-like malaise, each rated on a Likert scale from 0 to 4 for both frequency and severity. A symptom is considered positive if it is rated at least “moderate” in severity and occurs at least “half the time” during the past 6 months (53). DSQ-PEM has been validated across multiple languages and applied in post-COVID populations (54, 55). Its main advantages are standardized administration and broad applicability in epidemiological surveys and case definitions (56). However, as a self-report tool, it remains subjective and susceptible to recall bias.

### 4.2 Two-day cardiopulmonary exercise test (2-day CPET)

The 2-day CPET is an objective physiological test considered the most objective and reproducible measure of PEM (57). It measures cardiopulmonary performance (VO_2_max, workload, anaerobic threshold) on two consecutive days (58). Unlike healthy individuals, ME/CFS patients typically show a reduction of ≥8%−15% in VO_2_max or workload on the second day, reflecting impaired recovery and abnormal post-exertional physiology (59). This reproducible finding has been consistently validated in ME/CFS and replicated in long-COVID patients (51, 60). The strengths of 2-day CPET are its objectivity and strong empirical reproducibility, making it a valuable endpoint in clinical research (61). However, it is resource-intensive, requires specialized equipment and trained staff, and imposes a substantial burden on patients, which may limit its feasibility in routine clinical practice (58).

### 4.3 Functional capacity scale (FUNCAP)

The FUNCAP is a recently developed, patient-informed questionnaire designed to assess post-exertional functional decline in ME/CFS (50). It comprises a concise set of items evaluating activity reduction, physical exhaustion, and recovery delay, each scored on a 0–10 scale. Higher scores reflect greater functional impairment. As a newly introduced tool, FUNCAP has shown promise for capturing PEM with minimal patient burden, offering advantages in brevity, rapid administration, and potential scalability for large-cohort studies. However, current evidence is limited to its initial development and validation study, and further psychometric testing across diverse populations is required to establish diagnostic thresholds and comparability for research applications (50).

Table 2 summarizes the key assessment tools for PEM in ME/CFS, while detailed methodological and psychometric information is provided in Supplementary Table S2.

**Table 2:** Scales assessing PEM commonly used in ME/CFS.

**Scale**	**Content of the assessment**	**Advantages**	**Limitations**
DSQ-PEM	Six items, three dimensions (frequency, severity, duration of post-exertional symptoms); 6-month recall	Standardized, validated across cohorts; widely used in ME/CFS and post-COVID populations	Subjective self-report; recall bias; limited sensitivity to short-term changes
2-day CPET	Two sessions (two consecutive days); measures VO_2_max, workload, and anaerobic threshold decline; objective evaluation of post-exertional function	Objective and reproducible; best-validated physiological measure of PEM; strong empirical evidence	Resource-intensive; requires specialized equipment and trained staff; high patient burden
FUNCAP	Three domains (activity reduction, physical exhaustion, recovery delay); assesses post-exertional functional decline	Brief and patient-informed; low burden; scalable for large-cohort monitoring	New tool; limited psychometric validation; diagnostic thresholds not established

See Supplementary Table S2 for detailed characteristics.

## 5 Cognitive impairment-related assessment tools

Cognitive impairment is a characteristic manifestation of ME/CFS, mainly involving deficits in memory, attention, and information processing that significantly affect learning, occupational performance, and daily functioning (62). Braamse and colleagues reported clinically significant fatigue improvement in 63.2% of ME/CFS patients following cognitive behavioral therapy (CBT) (63), suggesting a close association between fatigue and cognitive dysfunction. Therefore, cognitive assessment is essential for both diagnosis and therapeutic evaluation in ME/CFS, providing standardized and practical measures of cognitive performance.

Cognitive assessment tools are generally classified as subjective or objective. Subjective measures rely on patient self-report to capture perceived difficulties across multiple domains, such as the montreal cognitive assessment (MoCA), the cognitive failure questionnaire (CFQ), and the everyday memory questionnaire (EMQ). Objective tools are performance-based and evaluate specific cognitive functions like attention, processing speed, and memory. They are often combined with self-reported measures to provide a comprehensive cognitive profile, including the trail making test (TMT) and the Wechsler memory scale-third edition (WMS–III) (64).

### 5.1 Subjective cognitive measures

#### 5.1.1 Montreal cognitive assessment (MoCA)

The MoCA, developed by Nasreddine as an enhanced alternative to the mini-mental state examination (MMSE), is a brief screening tool for mild cognitive impairment (MCI) with superior sensitivity and specificity (65). It includes 11 tasks across eight domains: attention and concentration, executive function, short-term memory, language, visuoconstruction, abstraction, calculation, and orientation (66). The total score is 30, with ≥26 considered normal and < 26 indicating cognitive impairment (67). Cut-offs of 21–22 and 19–20 are often used for MCI and Alzheimer's disease, respectively (68). Given its high sensitivity, the MoCA has been applied in ME/CFS to assess cognitive dysfunction. For instance, Murga et al. (66) used it to help differentiate cognitive impairment associated with ME/CFS. However, its performance is strongly influenced by education level, and its diagnostic specificity for dementia (69).

#### 5.1.2 Cognitive failures questionnaire (CFQ-25)

The CFQ-25, developed by Broadbent et al. (70), is a 25-item self-report tool designed to assess perceptual, memory and motor lapses in daily life. It has been widely applied in psychiatry and behavioral research, and is regarded as a useful measure of everyday cognitive errors (71). Each item is rated on a five-point Likert scale (0–4), producing a total score of 0–100, with higher scores indicating more frequent cognitive failures and greater subjective impairment (72). Attree et al. (73) later used the CFQ-25 to evaluate the frequency of perceptual, memory, and motor errors in ME/CFS when exploring psychosocial correlates of cognitive dysfunction. The CFQ-25 provides a global index of subjective cognitive difficulties linked to psychological factors but shows limited correspondence with objective performance, and its clinical interpretation should be cautious (74).

#### 5.1.3 Everyday memory questionnaire (EMQ)

The EMQ, developed by Sunderland et al. (75), is a 28-item self-report tool designed to assess everyday memory difficulties across five domains: retrieval, task monitoring, conversation monitoring, spatial memory, and active memory (76). Each item is rated on a nine-point scale from 1 (not at all in the past 6 months) to 9 (more than once a day), with higher scores indicating greater cognitive impairment. The EMQ has been used to evaluate cognitive dysfunction in ME/CFS when examining the relationship between subjective complaints and objective neuropsychological performance (77). It demonstrates good sensitivity for detecting mild memory problems in healthy populations and can be applied in both children and adults (78). However, its positive predictive value for identifying pathological memory disorders is limited, and it should not be used as a diagnostic tool (79).

Although numerous subjective cognitive measures exist, most have been rarely applied or not yet validated in ME/CFS research, except for the MoCA. Further development and validation of such tools for ME/CFS are warranted.

Table 3 summarizes the available subjective cognitive measures, while Supplementary Table S3 provides detailed information on their psychometric properties, validation evidence, and clinical applicability.

**Table 3:** Subjective cognitive measurement tools commonly used in ME/CFS.

**Scale**	**Content of the assessment**	**Advantages**	**Limitations**
MoCA	11 items, eight domains (attention and concentration, executive function, short-term memory, language, visuoconstruction, abstraction, calculation, and orientation); total 30 (≥26 normal)	Widely used; multidimensional; high sensitivity and specificity; suitable for screening and efficacy assessment	Education-dependent; limited diagnostic specificity; cannot identify underlying etiology of cognitive impairment
CFQ	25 items; assesses perceptual, memory, and motor lapses over past 6 months (Likert 0–4; total 0–100)	Reliable internal consistency; assesses everyday cognitive lapses; applicable in ME/CFS research	Subjective; influenced by psychological factors; limited correspondence with objective cognitive performance
EMQ	28 items, five domains (retrieval, task monitoring, conversation monitoring, spatial, active memory); 1–9 frequency-based scale	Sensitive to mild memory difficulties; applicable across age groups; captures everyday memory variations consistently	Low predictive validity for pathological memory loss; not suitable for diagnostic use

See Supplementary Table S3 for detailed characteristics.

### 5.2 Objective cognitive assessment instruments

#### 5.2.1 Trail making test (TMT)

The TMT, developed by Reitan et al. (80), is among the most widely used neuropsychological tools in both clinical and research settings and is frequently applied in ME/CFS studies (81). The test comprises two parts, A and B. In TMT-A, participants connect 25 numbered circles in sequence, whereas in TMT-B they alternately connect numbers (1–13) and letters (A–L). The time required to complete each task reflects information-processing speed and executive functioning, with longer completion times indicating poorer performance (82). Kujawski et al. (83) applied the TMT to assess cognitive function in ME/CFS patients and to evaluate the effects of whole-body cryotherapy combined with static stretching. The TMT is simple, quick to administer, and sensitive to executive dysfunction, making it suitable for clinical screening. However, it lacks specificity in differentiating underlying executive processes and provides only a crude, single-metric outcome, limiting its diagnostic precision (84).

#### 5.2.2 Wechsler memory scale-third edition (WMS-III)

The WMS-III, developed by Wechsler in 1997 in the United States, is one of the most widely used neurocognitive batteries in clinical practice (85). It assesses six cognitive domains: verbal comprehension, perceptual organization, processing speed, working memory, auditory memory and visual memory (86). Robinson et al. examined the cognitive manifestations of ME/CFS using the standardized WMS-III and the abbreviated Wechsler abbreviated scale of intelligence (WASI) to derive an overall intelligence quotient (IQ) encompassing vocabulary comprehension, visuoconstruction, verbal reasoning, and nonverbal deductive reasoning. They also used WMS-III subtests, including symbol search, digit symbol coding, digit span, and the family pictures test, to evaluate verbal memory, visual memory, working memory, and psychomotor speed (64). The WMS-III is efficient, practical, and capable of assessing both short- and long-term memory. However, it provides a relatively limited evaluation of broader cognitive domains and is influenced by cultural background, educational level, and examiner variability, which may introduce measurement bias (87).

#### 5.2.3 Other objective cognitive measures

In ME/CFS research, subjective scales are most commonly used to assess cognitive impairment, however, the integration of objective cognitive measures alongside subjective assessments has gained increasing attention. Given the wide range of available objective tools and the absence of standardized testing protocols, only the most frequently applied instruments are summarized here, including the TMT, the WMS-III, the Toulouse-Piéron Test (TP) (66), and the stroop color and word test (Stroop) (88). Most cognitive measurement tools, both subjective and objective, have not yet undergone sufficient psychometric validation in ME/CFS populations, warranting further reliability and applicability studies.

Table 4 summarizes the principal objective cognitive assessment tools, while Supplementary Table S4 provides detailed information on their psychometric properties, validation evidence, and clinical applicability.

**Table 4:** Objective cognitive measurement tools.

**Scale**	**Content of the assessment**	**Advantages**	**Limitations**
TMT	Two parts (A/B): TMT-A connects numbers 1–25; TMT-B alternates numbers (1–13) and letters (A–L). Assesses processing speed and executive function.	Sensitive to executive dysfunction; simple, intuitive, and quick; easy to administer	Low specificity; cannot distinguish multiple executive processes; single and coarse outcome metric
WMS-III	Neurocognitive battery assessing six domains: verbal comprehension, perceptual organization, processing speed, working memory, and auditory and visual memory.	Efficient, practical; assesses both short- and long-term memory	Limited coverage of broader cognitive domains; influenced by culture and education; examiner variability may introduce measurement bias
TP	Assesses selective/sustained attention, perception, and processing speed; outputs include dispersion index (higher = worse) and work efficiency (lower = worse) (89).	Suitable for screening and treatment evaluation; classic test of attentional performance (66)	Influenced by age and education; reflects impairment only; non-diagnostic (90)
Stroop	Color–word interference task assessing processing speed, attention control, and response inhibition (91).	Widely applicable; quick to administer; suitable for elderly or easily fatigued individuals	Influenced by age, education, vision, and sleep; not diagnostic for cognitive impairment (92–94)

See Supplementary Table S4 for detailed characteristics.

## 6 Assessment of sleep status

Patients with ME/CFS often experience difficulty falling asleep and disrupted circadian rhythms, potentially linked to central hyperadrenergic activity or hypocapnia (95). Kallestad et al. (96) reported that alleviating insomnia severity could reduce fatigue, suggesting that insomnia may act as a maintenance factor for chronic fatigue. Thus, evaluating sleep-related parameters represents a theoretically sound and clinically relevant approach to understanding fatigue in ME/CFS.

Sleep assessment tools are generally classified as subjective or objective. Objective measures more accurately distinguish sleep from wakefulness, whereas subjective scales capture the perceived impact of sleep disturbances on daily functioning (97). As varying measurement approaches may yield different prevalence estimates of sleep disorders within the same cohort (98, 99), selecting appropriate and validated sleep instruments is crucial in ME/CFS research.

### 6.1 Objective sleep measurement tools

#### 6.1.1 Polysomnography (PSG)

Polysomnography (PSG), also known as a sleep electroencephalogram, is considered the gold standard for objective assessment of sleep and sleep-wake rhythms and remains the most widely used objective tool for evaluating sleep parameters in ME/CFS research (100–102). It provides detailed data on sleep architecture, including total sleep time, sleep latency, sleep efficiency, frequency and duration of awakenings, and the proportion of non-rapid eye movement (NREM) and rapid eye movement (REM) sleep (103). PSG can aid in diagnosing insomnia phenotypes and in evaluating the effectiveness of therapeutic interventions both within and outside laboratory (104). PSG records multiple physiological signals from various sensors placed on the body, which are amplified and converted into electrical outputs for analysis. In addition to the electroencephalogram (EEG), standard PSG monitoring includes more than 10 physiological channels, such as electrocardiogram (ECG), electromyogram (EMG), and electro-oculography (EOG) (105). Decker et al. (106) conducted overnight PSG assessments in ME/CFS patients (106), while Neu et al. (107) compared spectral power ratios between ME/CFS and primary insomnia during slow-wave sleep. Despite its comprehensive and accurate evaluation capabilities, PSG is costly, requires specialized technical expertise, and imposes substantial procedural and environmental constraints on participants.

#### 6.1.2 Actigraphy (ACT)

Actigraphy (ACT) offers a convenient, non-invasive, and quantitative approach for objectively assessing sleep-wake patterns. It is particularly useful for infants, young children, and critically ill patients. The device, typically worn on the non-dominant wrist, records rest-activity cycles through motion sensors to infer sleep and wake states (108). Russell and colleagues applied actigraphy to differentiate sleep from wakefulness and to predict next-day fatigue in ME/CFS patients (109). Compared with PSG, actigraphy provides greater comfort, minimal interference with natural sleep, and enables long-term monitoring. However, as it infers sleep from movement rather than neural activity, it cannot distinguish immobile wakefulness from true sleep and may be influenced by comorbidities or motor disorders. To enhance accuracy, actigraphy is often combined with sleep diaries (110).

Table 5 summarizes the objective sleep assessment tools, while Supplementary Table S5 details their psychometric properties, validation evidence, and clinical applicability.

**Table 5:** Objective sleep measurement tools commonly used in ME/CFS.

**Scale**	**Content of the assessment**	**Advantages**	**Limitations**
PSG	Records multi-channel biosignals (EEG, ECG, EMG, EOG) to analyze total sleep time, latency, efficiency, awakenings, and NREM/REM proportions	Gold-standard; comprehensive and accurate; enables detailed sleep-stage analysis	Expensive; requires trained technicians; limited accessibility; may disturb natural sleep
ACT	Wrist-worn motion sensor monitoring rest–activity cycles to infer sleep and wake states	Non-invasive; inexpensive; suitable for long-term monitoring; minimal sleep interference	Cannot differentiate quiet wakefulness from true sleep; influenced by motor disorders; requires concurrent sleep diary

See Supplementary Table S5 for detailed characteristics.

### 6.2 Subjective sleep scales

#### 6.2.1 The Pittsburgh sleep quality index (PSQI)

The Pittsburgh sleep quality index (PSQI), developed by Buysse et al., is one of the most widely used self-rated instruments for assessing sleep quality and disturbances in ME/CFS research. It evaluates seven domains of sleep over the past month: subjective sleep quality, sleep latency, sleep duration, habitual sleep efficiency, sleep disturbances, use of sleep medication, and daytime dysfunction (111–113). The scale comprises 19 items, each rated on a 0–3 Likert scale (0 = no difficulty, 3 = severe difficulty), yielding a total score ranging from 0 to 21; higher scores indicate poorer sleep quality, and a global score ≥5 denotes significant sleep disturbance.

Castro-Marrero et al. (114) used the PSQI to assess sleep quality and its impact on quality of life in Spanish ME/CFS patients, while Wei et al. (115) applied it to examine insomnia severity and circadian rhythm alterations associated with serum factors in ME/CFS. Although the PSQI reflects subjective sleep perception and cannot determine the specific etiology of sleep disturbance (116), it assesses both qualitative and quantitative aspects of sleep, differentiates transient from persistent insomnia, and demonstrates strong concordance with PSG findings (117). Owing to its practicality, psychometric validity, and broad applicability, the PSQI remains a reliable and versatile instrument for clinical and research evaluation of sleep in ME/CFS.

#### 6.2.2 Epworth sleepiness scale (ESS)

The Epworth sleepiness scale (ESS), developed by the Epworth Sleep Research Center in Australia, is a self-rated instrument for the subjective assessment of excessive daytime sleepiness (EDS) and has been used alongside the PSQI in ME/CFS studies (118). It evaluates sleep propensity across eight daily situations, including reading, watching television, attending meetings, driving for 1 h during the day, lying down to rest in the afternoon, talking to others, sitting quietly after meals, and driving in traffic or waiting at a light. Each item is rated on a 0–3 Likert scale (0 = never, 3 = often), yielding a total score ranging from 0 to 24; scores of 0–9 indicate normal alertness, 10–15 suggest possible sleepiness, and 16–24 denote excessive sleepiness (119). Cameron (120) used the ESS to assess daytime sleepiness in ME/CFS patients while validating the Flinders fatigue scale as a measure of daytime fatigue. The ESS offers accurate scoring, simple self-administration, and broad applicability, making it one of the most practical instruments for evaluating daytime sleepiness.

#### 6.2.3 Other methods of assessing sleep status

PSG and the PSQI are the most commonly used assessment tools in ME/CFS clinical research. However, many studies have also used additional instruments to evaluate sleep quality from different perspectives, most of which are subjective measures. Commonly used alternatives include the insomnia severity index (ISI) (121) and the sleep diary (122), among others.

Table 6 summarizes the subjective sleep assessment tools, while Supplementary Table S6 provides detailed information on their psychometric properties, validation evidence, and clinical applicability.

**Table 6:** Subjective sleep measurement tools commonly used in ME/CFS.

**Scale**	**Content of the assessment**	**Advantages**	**Limitations**
PSQI	Assesses seven domains: subjective sleep quality, sleep latency, sleep duration, habitual sleep efficiency, sleep disturbances, use of sleep medications and daytime sleep disturbances	Captures both sleep quality and quantity; distinguishes transient vs. persistent disorders; consistent with PSG	Subjective bias; cannot identify causes; limited for long-term (>1 month) evaluation.
ESS	Assesses the patient's tendency to fall asleep during eight common daily situations.	Simple, quick, and reliable; suitable for screening	Evaluates only daytime sleepiness; not diagnostic for sleep disorders
ISI	Self-rated assessment of insomnia type, severity, and daily impact during the past month (123)	Brief, targeted, and useful for screening or outcome tracking (124)	Emotionally influenced; lacks ME/CFS-specific validation
Sleep diary	Daily self-reporting of nighttime sleep and daytime performance (125)	Easy, low-burden, and provides better accuracy than recall questionnaires (126)	Highly subjective; low reliability; auxiliary only to objective tools (112)

See Supplementary Table S6 for detailed characteristics.

## 7 Pain-related assessment tools

ME/CFS is frequently accompanied by pain symptoms such as headache, sore throat, and muscle or joint pain, which often worsen following exertion (127). Pain is recognized as a key accompanying symptom (128). Accordingly, pain assessment tools are essential in both clinical evaluation and research. Commonly used instruments include the visual analog scale (VAS) (129), the numeric rating scale (NRS) (130), and the McGill pain questionnaire (MPQ) (131).

### 7.1 Visual analog scale (VAS)

The VAS is one of the most commonly used unidimensional instruments for assessing pain intensity. It consists of a 100-mm horizontal line anchored by “no pain” at one end and “severe pain” at the other, where patients indicate their perceived pain level by marking a point along the line (132, 133). Kempke et al. (134) used the VAS to evaluate pain severity in a study examining the association between self-critical or maladaptive perfectionism and ME/CFS. The VAS is simple, quick, sensitive, and relatively objective, making it suitable for assessing pain intensity and comparing pre- and post-treatment effects. However, its use requires a certain level of abstract reasoning, and scores are not directly comparable across individuals (135).

### 7.2 Numeric rating scale (NRS)

The NRS is a numerical adaptation of the VAS that asks patients to rate their pain intensity on an 11-point scale from 0 to 10, where 0 indicates no pain, 1–3 mild pain, 4–6 moderate pain, and 7–10 severe pain, with 10 representing the most intense pain (136). Thompson et al. (137) used the NRS to evaluate pain severity in ME/CFS patients when examining the association between activity pacing and symptom fluctuation. The NRS is simple, intuitive, and widely used, showing strong concordance with the VAS. However, it requires adequate verbal comprehension and understanding of numerical concepts, and may be influenced by linguistic or cognitive factors, leading to slightly lower sensitivity and accuracy (138, 139).

### 7.3 McGill pain questionnaire (MPQ)

The MPQ, developed by Melzack (140), is a classic multidimensional instrument for assessing pain across three domains: sensory, affective, and evaluative. It includes 78 descriptors grouped into four categories and 20 subclasses, each arranged in order of increasing intensity. Participants select the words that best describe their pain; if none apply, they may skip the group (141). The MPQ yields three indices: the pain rating index (PRI, calculated from the ordinal values of chosen descriptors), the number of words chosen (NWC), and the present pain intensity (PPI, rated from 0 = no pain to 5 = excruciating pain). Mckay et al. (142) used the MPQ in a quasi-experimental study exploring the relationship between ME/CFS and fibromyalgia to assess pain frequency and intensity. The MPQ is sensitive to treatment-related changes and useful for distinguishing nociceptive and neuropathic pain (143). However, it is lengthy, literacy-dependent, time-consuming, and influenced by demographic factors such as gender and race.

### 7.4 Other pain assessment scales

In addition to the instruments described above, several other tools have been used to evaluate pain in ME/CFS, including the brief pain inventory (BPI) (144) and the pain catastrophizing scale (PCS) (145).

Table 7 summarizes the pain-related assessment tools, while Supplementary Table S7 provides detailed information on their psychometric properties, validation evidence, and clinical applicability.

**Table 7:** Pain measurement tools commonly used in ME/CFS.

**Scale**	**Content of the assessment**	**Advantages**	**Limitations**
VAS	Assessment of current pain intensity using a 100-mm line from “no pain” to “severe pain”	Simple; quick; sensitive; quantitative; allows fine-grained measurement of subjective pain; suitable for pre- and post-treatment comparison	Requires abstract reasoning; unidimensional; not comparable across subjects
NRS	Rating of pain intensity on an 11-point scale (0 = no pain, 10 = worst pain)	Easy to understand; simple; intuitive; high consistency with VAS; widely used in clinical settings	Requires verbal and numerical understanding; affected by language and cognition; lower sensitivity and accuracy
MPQ	Multidimensional assessment of pain (sensory, affective, evaluative dimensions); 78 descriptors in 20 groups	Sensitive to treatment effects; distinguishes nociceptive and neuropathic pain; comprehensive evaluation	Lengthy; requires literacy; time-consuming; influenced by gender and ethnicity
BPI	Assessment of pain intensity and interference in seven domains: activity, mood, walking, relations, sleep, enjoyment (146, 147)	Multidimensional; quick; easy to administer; applicable across populations (148)	Limited validation in ME/CFS; cannot diagnose neuropathic pain
PCS	Assessment of pain catastrophizing across three dimensions: rumination, magnification, and helplessness (149)	Simple; self-reported; convenient; psychometrically validated (150)	Indirect measure of pain fear; subjective; prone to response bias

See Supplementary Table S7 for detailed characteristics.

## 8 Psychological state assessment scales

Psychological comorbidities, particularly anxiety and depression, are common in ME/CFS, with prevalence rate reaching 42.2 and 33.3%, respectively (151). Therefore, evaluating psychological status is essential in both clinical and research settings. Commonly used instruments include examiner-rated and self-rated scales. Examiner-rated tools frequently applied in ME/CFS research include the hospital anxiety and depression scale (HADS), the Hamilton anxiety rating scale (HAMA), and the Hamilton depression rating scale (HAMD). Self-rated scales such as the self-rating anxiety scale (SAS), the self-rating depression scale (SDS), and the Beck depression inventory (BDI) are also widely used.

### 8.1 Hospital anxiety and depression scale (HADS)

The HADS, developed by Zigmond and Snaith (152), is a reliable and valid instrument designed to assess symptoms of anxiety and depression in general medical populations. It consists of two subscales, the anxiety scale (HADS-A) and the depression scale (HADS-D), comprising a total of 14 items, with seven items for each domain. Each item is rated on a four-point Likert scale from 0 to 3, yielding a total score ranging from 0 to 42. A score of 9 or above is generally considered indicative of anxiety or depression (153). Loades and colleagues used the HADS as an outcome measure in a cross-sectional epidemiological study of adolescents with ME/CFS presenting with comorbid anxiety and depression (154). The HADS is independent of somatic symptoms and demonstrates strong discriminative validity for psychological distress in ME/CFS (155). However, its ability to differentiate clearly between anxiety and depression constructs remains uncertain, and psychometric validation specific to ME/CFS populations is still limited, suggesting that reliability may vary across settings.

### 8.2 Hamilton anxiety rating scale (HAMA) and Hamilton depression rating scale (HAMD)

The HAMA and HAMD, both developed by Hamilton (156, 157), are widely used examiner-rating instruments for evaluating anxiety and depression. The HAMA consists of 14 items that assess psychological and somatic symptoms of anxiety (158), and demonstrates good reliability and validity in reflecting both symptom severity and treatment (159). The 17-item version of the HAMD (HAMD-17) is the most widely used in ME/CFS studies and covers anxiety (psychological and somatic), somatic symptoms (gastrointestinal and general), depression, and insight (160). Both scales use a 0–4 scoring system, with higher scores indicating greater symptom severity and a threshold score of eight suggesting clinical significance (161).

Tingting et al. (162) used the HAMA to assess anxiety and the SDS to evaluate depression in a study on spaced ginger moxibustion for ME/CFS. Although some items overlap, including depressive mood, somatic anxiety, gastrointestinal symptoms, and insomnia, which may blur the distinction between anxiety and depression (163). However, factor analyses support the ability of both scales to detect symptom changes and treatment effects. Owing to their long history of clinical use and demonstrated psychometric robustness, the HAMA and HAMD remain among the most established tools for assessing psychological status in ME/CFS (164).

### 8.3 Self-rating anxiety scale (SAS) and self-rating depression scale (SDS)

The SAS and SDS, both developed by Zung in 1971, are 20-item, four-point self-report instruments with similar structures and scoring methods (165). The SAS assesses anxiety symptoms aligned with major U.S. psychiatric diagnostic criteria and includes 15 negatively and five positively worded items (166). Although primarily designed for anxiety, it also reflects depressive tendencies due to overlapping affective components (167). The SDS, comprising 10 negative and 10 positive items, focuses primarily on emotional and somatic symptoms of depression (168, 169).

Meng et al. (170) employed both the SAS and the SDS as secondary outcome measures to evaluate the efficacy of different pressure cupping interventions for ME/CFS, alongside fatigue and sleep scales. When used together, these two instruments provide a rapid, reliable, and comprehensive evaluation of anxiety and depression symptoms, as well as their severity and temporal changes in ME/CFS. When combined with the fatigue assessment tools, they can help differentiate ME/CFS from primary anxiety or depressive disorders, although their accuracy may be limited in individuals with lower literacy or cognitive ability.

### 8.4 Other psychological status assessment scales

In addition to the aforementioned scales, various tools have been employed in ME/CFS studies to assess psychological status, including the BDI, the symptom checklist-90 (SCL-90), the center for epidemiologic studies depression scale (CES-D), and the general health questionnaire (GHQ), among others. Recognizing the inherent subjectivity and time sensitivity of psychometric measures, both clinical and research protocols often combine multiple instruments or integrate self-report with examiner-rated assessments to minimize bias and enhance accuracy.

Table 8 summarizes the psychological state assessment scales, while Supplementary Table S8 provides details on their psychometric properties, validation evidence, and clinical applicability.

**Table 8:** Psychological status measurement tools commonly used in ME/CFS.

**Scale**	**Content of the assessment**	**Advantages**	**Limitations**
HADS	Assesses anxiety and depression simultaneously in general medical populations.	Examiner-rated scale; unaffected by somatic symptoms; good discriminative validity for psychological distress in ME/CFS	Limited evidence of psychometric robustness in ME/CFS; difficulty distinguishing anxiety from depression; requires trained raters
HAMA	Evaluates psychological and somatic symptoms of anxiety.	Reliable and valid; reflects symptom severity and treatment response; widely used in clinical trials	Overlaps with depression-related items; requires professional administration; limited ME/CFS-specific validation
HAMD	Assesses depression severity, including anxiety (psychological and somatic), somatic symptoms (general and gastrointestinal), depression, and insight domains	Sensitive to symptom changes and treatment effects; extensively applied in ME/CFS and psychiatric research	Item overlap with HAMA; time-consuming; may not clearly separate anxiety from depression constructs
SAS	Self-rated affective symptoms of anxiety during the past week.	Simple, rapid, widely used; captures both anxiety severity and treatment changes	Subjective self-report; may overestimate comorbidity with depression; less accurate in low-literacy populations
SDS	Self-rated emotional and somatic symptoms of depression over the past week.	Convenient, reliable, and sensitive to treatment-related changes; often combined with fatigue scales in ME/CFS research	Non-diagnostic; limited accuracy in individuals with cognitive or literacy difficulties
BDI (171)	Assesses subjective feelings and cognitive-affective symptoms of depression.	Reliable and valid; sensitive to clinical change; distinguishes depressive symptom severity (172)	Does not differentiate depression subtypes; not suitable for illiterate or poorly educated groups
CES-D (173)	Evaluates frequency and severity of depressive symptoms, including mood and interpersonal functioning.	Highly sensitive for screening depressive symptoms; suitable for large-scale or epidemiologic studies (174)	Cannot diagnose depression or monitor symptom severity; self-report bias
SCL-90 (175)	Assess the patient's current/last week somatization, Measures current or recent symptoms of depression, anxiety, hostility, phobia, and psychoticism (176).	Covers multiple psychological dimensions; allows broad mental health screening	Highly subjective; not diagnostic; limited clinical specificity for ME/CFS
GHQ (177)	Screens for depression, anxiety, insomnia, somatic symptoms, and social dysfunction.	High sensitivity and specificity; useful for population screening and epidemiological surveys (178)	Designed for general mental-health screening rather than ME/CFS-specific assessment; unsuitable for tracking treatment effects

See Supplementary Table S8 for detailed characteristics.

## 9 Orthostatic intolerance (OI) assessment tools

Orthostatic intolerance (OI) has been recognized as a key diagnostic feature of ME/CFS since the 2015 National Academy of Medicine (NAM) criteria (179). The prevalence of OI symptoms is ~82% in adults and 96% in adolescents with ME/CFS, making it an important distinguishing characteristic from other disorders. OI encompasses a spectrum of symptoms induced by upright posture, including delayed orthostatic hypotension, reflex syncope, and postural tachycardia syndrome (POTS) (180). These manifestations are primarily associated with reduced cardiovascular and cerebral blood flow and activation (181). Assessment tools for OI include objective measures such as changes in blood pressure (BP), heart rate (HR), and cerebral blood flow during provocation, as well as subjective evaluations obtained through self-rated or examiner-rated questionnaires.

### 9.1 Objective OI assessment tools

#### 9.1.1 Head-up tilt test (HUT)

The head-up tilt test (HUT), first introduced into clinical use by Kenny et al. (182), was designed to assess physiological responses to upright posture. In this procedure, the subject lies supine on a tilt table for 10 min under continuous BP and ECG monitoring. BP and HR are measured every 5 min (three times), then every minute for the first 5 min after tilting to 70 °, and subsequently every 5 min for 30 min. If dizziness, syncope, or loss of consciousness occurs, measurements are repeated every 30 s and the test terminated if the subject becomes unconscious (183). van Campen et al. (184) reported abnormal cerebral blood flow reduction in about 90% of ME/CFS patients during HUT, along with hemodynamic, HR, BP, and end-expiratory CO_2_ changes. HUT is widely used in ME/CFS studies to evaluate orthostatic hypotension, chronic orthostatic intolerance, and unexplained syncope, aiding in the identification of OI as a comorbid feature (185). However, it requires professional supervision and may yield false-positive (186).

#### 9.1.2 Active 10-min standing test (AST)

The active 10-min standing test (AST), first applied by Ash-Bernal et al. (187), was used to assess autonomic and vestibular responses to upright posture in ME/CFS. During the test, patients stand quietly for 10 min without moving their feet while HR and BP are continuously monitored. The test is considered failed if the patient cannot maintain the standing position and discontinues the test because of palpitations, dizziness, pallor, fatigue, weakness, lightheadedness, tremor, or nausea (188). Miwa et al. (189) evaluated AST and postural orthostatic tachycardia in ME/CFS, demonstrating that autonomic imbalance contributes to OI. Compared with the HUT, AST is less frequently used in ME/CFS studies because of its lower specificity, sensitivity, and positive predictive value (190).

Table 9 summarizes the objective measurement tools for OI, while Supplementary Table S9 provides details on their psychometric properties, validation evidence, and clinical applicability.

**Table 9:** OI objective measurement tools commonly used in ME/CFS.

**Scale**	**Content of the assessment**	**Advantages**	**Limitations**
HUT	Measure BP, HR, or cerebral blood flow by changing subject's position (from lying to tilted)	Simple, safe, non-invasive; effective for diagnosing vasovagal syncope; high specificity and sensitivity	Requires professional operation; not suitable for all patients; false-positive results possible
AST	Stand for 10 min without changing foot position and take measurements such as HR and BP during the standing test	Convenient and easy to perform; detects sympathetic responses to upright posture	Low specificity, sensitivity, and positive predictive value; prone to misinterpretation during administration and interpretation

See Supplementary Table S9 for detailed characteristics.

### 9.2 Subjective evaluation scales for OI

#### 9.2.1 Composite autonomic symptom score-31 (COMPASS-31)

The composite autonomic symptom score-31 (COMPASS-31), developed by Sletten et al. (191), is a simplified version of the original 84-item COMPASS questionnaire. It includes six domains, namely orthostatic intolerance, vasomotor, secretomotor, pupillary motility, gastrointestinal transport and bladder function, with total scores ranging from 0 to 100, where higher scores indicate more severe autonomic symptoms. COMPASS-31 is useful for identifying patients with suspected autonomic dysfunction, monitoring symptom severity, and evaluating treatment response, showing good discrimination of autonomic abnormalities in ME/CFS (192, 193). Martin et al. (194) applied COMPASS-31 to assess autonomic function in fibromyalgia and ME/CFS, correlating the results with biomarkers of gut barrier dysfunction and bacterial translocation. Despite increasing recognition of OI as a diagnostic feature of ME/CFS, related assessment tools remain limited, and further validation of OI-specific instruments is needed.

#### 9.2.2 Orthostatic grading scale (OGS)

The orthostatic grading scale (OGS), developed by Schrezenmaier et al. in 2005 (195), is a self-report questionnaire designed to assess symptoms of OI caused by orthostatic hypotension (195). It comprises five items, including frequency, severity, triggering situations, standing time, and impact on daily activities, with each item scored from 0 to 4 to yield a total score ranging from 0 to 20, where higher scores indicate more severe autonomic dysfunction (196). OGS has been used by Costigan and Jones and their colleagues to evaluate orthostatic symptoms in ME/CFS (196, 197).

Table 10 summarizes the subjective evaluation scales for OI, while Supplementary Table S10 provides details on their psychometric properties, validation evidence, and clinical applicability.

**Table 10:** OI subjective measurement tools commonly used in ME/CFS.

**Scale**	**Content of the assessment**	**Advantages**	**Limitations**
COMPASS-31	Evaluates severity of upright intolerance across six domains: orthostatic intolerance, vasomotor, secretomotor, pupillomotor, gastrointestinal, and bladder function.	Convenient self-report; tracks autonomic severity and treatment response; sensitive to autonomic deficits in ME/CFS	Not ME/CFS-specific and partly subjective, limiting precision for disease-specific assessment
OGS	Assess frequency, severity, triggering scenarios, duration of standing, and impact on daily activities of orthostatic symptoms.	Rapid, reliable screening correlated with autonomic tests; simple to administer	Not validated in ME/CFS; useful only for preliminary screening, not diagnostic for OI

See Supplementary Table S10 for detailed characteristics.

## 10 Multi-dimensional health scales

In modern healthcare, the definition of health has expanded beyond the absence of disease to encompass overall wellbeing, making health-related quality of life (HRQoL) an essential outcome measure. The ultimate goal of medical treatment is not only to alleviate symptoms but also to enhance patients' physical, psychological, and social functioning. Evaluating and improving multidimensional health status during and after treatment is therefore particularly important for functional disorders such as ME/CFS (198). Commonly used multidimensional health assessment instruments include the 36-item short form health survey (SF-36), the abbreviated world health organization quality of life questionnaire (WHOQoL-BREF), and the EuroQol five dimensions questionnaire (EQ-5D).

### 10.1 36-item short form health survey (SF-36)

The SF-36 evolved from the earlier medical outcomes study short form (MOS SF) questionnaires first described by Stewart et al. (199), with the finalized 36-item version later developed by Ware and Sherbourne (200). It is one of the most widely used self-report instruments for evaluating health-related QoL across diverse populations. The SF-36 assesses physical, psychological, and social functioning, as well as overall health status, with total scores ranging from 0 to 100, where lower scores indicate poorer health (201). Kim et al. (202) reported that 30.9% of randomized controlled trials (RCTs) on ME/CFS employed the SF-36 to examine QoL outcomes. However, certain items may be overly sensitive and fail to distinguish moderate-to-severe ME/CFS cases, while the instrument's length may limit its practicality in large-scale surveys (203).

### 10.2 EuroQol five dimensions questionnaire (EQ-5D)

The EQ-5D, developed by the EuroQol Group in 2001, is a standardized instrument for measuring health status in clinical and population studies (204). Several versions exist, including EQ-5D-3L, EQ-5D-5L, and the youth version, EQ-5D-Y (205). The EQ-5D-3L comprises the descriptive system and the visual analog scale (EQ-VAS), with the descriptive system assessing five key dimensions, namely mobility, self-care, usual activities, pain or discomfort, and anxiety or depression, each rated at three levels: no problems, some problems, and extreme problems (206). The EQ-VAS records self-rated health on a 0–100 vertical scale, where 0 represents the worst and 100 the best imaginable health (207). To improve sensitivity and reduce ceiling effects, the EuroQol group developed the EQ-5D-5L, which expands each dimension to five response levels (208). Mapping studies have shown that the EQ-5D-5L provides greater reliability across different ages and genders (209). In ME/CFS research, Salonen et al. (27) applied the EQ-5D-3L to assess changes in health-related QoL after fecal microbiota transplantation, whereas Orji et al. (210) used the EQ-5D-5L in a cross-sectional study of Australian patients.

### 10.3 World health organization quality of life questionnaire (WHOQOL-BREF)

The WHOQOL-BREF (211), developed by the WHOQOL group as an abbreviated version of WHOQOL-100, is a cross-cultural instrument designed to assess QoL across diverse populations. It consists of 26 items covering four domains: physical health, psychological health, social relationships, and environment. Each item is rated on a five-point Likert scale ranging from “not at all” to “extremely,” and domain scores are calculated by averaging item values and multiplying by four. The weighted sum of domain scores provides the overall QoL score, with higher scores indicating better quality of life (212). Brittain et al. (213) applied WHOQOL-BREF to evaluate QoL among individuals with ME/CFS and their family members. Although it lacks the detailed assessment of 24 specific QoL facets included in the WHOQOL-100, its brevity and ease of administration make it suitable for clinical research and trials where time constraints or participant burden limit the use of longer instruments.

### 10.4 Other multidimensional health scales

In addition to the above instruments, the Nottingham health profile (NHP) and the sickness impact profile (SIP) have also been used in ME/CFS research. QoL scales are generally broad and nonspecific, mainly used for overall health assessment in clinical and research contexts. Few QoL tools are included in ME/CFS studies, and most capture symptoms that partly overlap with psychological, sleep, or functional domains. When selecting multidimensional health measures, it is important to consider not only overall health status but also the ability to reflect other relevant symptoms for a more comprehensive evaluation.

Table 11 summarizes the commonly used multi-dimensional health scales, while Supplementary Table S11 provides details on their psychometric properties, validation evidence, and clinical applicability.

**Table 11:** Multi-dimensional health scales commonly used in ME/CFS.

**Scale**	**Content of the assessment**	**Advantages**	**Limitations**
SF-36	Assesses HRQoL across eight domains: physical functioning, physical role, bodily pain, general health, vitality, social functioning, emotional role, and mental health.	Simple and widely applicable; enables comparison of physical, psychological, and social functioning among individuals	Fails to effectively distinguish moderate-to-severe ME/CFS cases; limited feasibility in large-scale surveys
EQ-5D	Describes health status across five dimensions: mobility, self-care, usual activities, pain/discomfort, and anxiety/depression	Reliable and specific; EQ-5D-5L provides improved accuracy and reduced ceiling effects	EQ-5D-3L shows low sensitivity to small health changes; EQ-5D-5L is more complex and time-consuming to administer
WHOQOL-BREF	Evaluates four HRQoL domains: physical, psychological, social relationships, and environment.	Brief and convenient; suitable for studies with time constraints or high respondent burden	Does not comprehensively assess 24 detailed QoL facets of the WHOQOL-100; prone to subjective bias
NHP (214)	Includes two parts: health questionnaire (six domains—physical activity, pain, social isolation, emotional response, energy, sleep) and personal life issues (seven aspects of daily life) (215).	High sensitivity; covers sleep and pain dimensions relevant to ME/CFS populations	The first part lacks a total score and full health evaluation; the second part is vague and less weighted
SIP	A 136-item tool assessing impairment in 12 dimensions, including occupation, interpersonal relationships, family management, and physical health (216).	Comprehensive coverage with well-defined and weighted indicators (217)	Time-consuming; requires patient communication to confirm that impairments are health-related

See Supplementary Table S11 for detailed characteristics.

## 11 Discussion

Behavioral assessments are essential in ME/CFS research and clinical management, covering fatigue, cognition, psychology, pain, sleep, orthostatic intolerance, and multidimensional health aspects. Recent developments have introduced multidimensional tools such as the Munich-Berlin symptom questionnaire (MBSQ), which systematically assesses PEM, cognitive dysfunction, and autonomic symptoms across different age groups (218). Similarly, the COVID-19 Yorkshire rehabilitation scale (C19-YRS), although originally developed for post-COVID syndrome, shares substantial symptom overlap with ME/CFS (219). These advances reflect the convergence between ME/CFS and post-infectious fatigue syndromes, highlighting the need for harmonized multidomain assessment tools.

The diversity of existing scales mirrors the heterogeneity of ME/CFS. Tools focusing solely on fatigue risk underdiagnosis or misdiagnosis, whereas excessive emphasis on secondary symptoms may reduce specificity. A comprehensive multidimensional approach therefore remains crucial. This review summarizes current tools and their psychometric properties to guide appropriate scale selection and promote the development of standardized, reliable instruments for ME/CFS and related post-infectious conditions.

### 11.1 The impact of different populations (children, older adults, and post-COVID ME/CFS) and individual differences on the selection and outcomes of assessment scales

Physiological and psychological characteristics that vary across age groups and populations can lead to distinct manifestations and assessment outcomes of ME/CFS symptoms. Children and older adults often present different symptom patterns and coping mechanisms, indicating that instruments designed for adults may not be fully applicable to these groups. For instance, children tend to exhibit greater emotional and behavioral disturbances, whereas older adults experience more pronounced physical decline (220). Furthermore, patients with post-COVID ME/CFS share many core symptoms with traditional ME/CFS but may present with more complex or overlapping mechanisms involving autonomic, inflammatory, and neurocognitive domains (221). These differences highlight the importance of using assessment tools with higher sensitivity and specificity to detect subtle variations across populations. Therefore, the development and validation of population-adapted or age-specific instruments are essential to ensure accurate symptom characterization and enhance the clinical and research applicability of ME/CFS assessments.

### 11.2 The importance of appropriate assessment tools in long-term management of ME/CFS

Selecting appropriate assessment tools is essential for the long-term management of ME/CFS. Validated instruments enable clinicians to accurately evaluate symptom severity and functional status, forming the basis for individualized treatment planning. Standardized tools also facilitate monitoring of symptom changes, allowing timely adjustment of therapeutic strategies and improvement of patients' overall health and functioning. In research, consistent use of validated measures supports reliable data collection and enhances comparability across clinical trials (222).

Although many existing scales demonstrate acceptable reliability and validity, most remain subjective and prone to observer or recall bias. Some studies rely solely on fatigue-related tools, which may increase measurement error. Combining multiple instruments and integrating subjective questionnaires with objective indicators, such as physiological or digital measures, can improve accuracy and reduce bias. Furthermore, factors such as test duration and patients' health status should be considered when selecting instruments to ensure feasibility, reproducibility, and clinical relevance.

### 11.3 The necessity for developing emerging assessment tools

As understanding of ME/CFS continues to advance, the limitations of existing instruments have become increasingly evident, especially in the context of post-COVID ME/CFS and other post-infectious syndromes. Developing novel assessment approaches is therefore essential to address unmet clinical and research needs. Future instruments should integrate recent clinical and neurobiological findings and adopt flexible, multidimensional frameworks capable of capturing both subjective experiences and objective indicators. For example, tools combining biomarker analysis with patient-reported outcomes may allow a more comprehensive evaluation of disease status. In addition, the growing use of digital health technologies, including mobile applications and wearable monitoring systems, provides opportunities for real-time assessment and longitudinal tracking of symptoms (223, 224). The development and validation of such innovative instruments will enhance diagnostic precision and support more individualized management strategies for patients with ME/CFS.

### 11.4 The standardization and normalization development model for ME/CFS symptom assessment

The standardization and normalization of ME/CFS symptom assessment are crucial for improving diagnostic accuracy and enhancing treatment outcomes. Establishing unified assessment standards and procedures can ensure consistency and comparability of results across healthcare and research settings, thereby facilitating longitudinal monitoring and inter-institutional collaboration. International cooperation and multicenter studies are particularly important, as they allow integration of data from diverse regions and populations to refine, validate, and update assessment tools (225). In parallel, advances in artificial intelligence and big data analytics offer opportunities to develop adaptive and personalized assessment systems capable of real-time evaluation and feedback based on individual symptom profiles. The integration of these technologies within standardized frameworks will further promote the objectivity, reproducibility, and clinical applicability of ME/CFS assessments, ultimately supporting precision diagnosis and evidence-based management.

### 11.5 Practical recommendations for selecting ME/CFS assessment scales

To facilitate the selection of appropriate instruments for clinical and research applications, Table 12 summarizes recommended and less recommended scales by symptom domain, considering their psychometric evidence, sensitivity, and applicability across populations.

**Table 12:** Practical recommendations for commonly used ME/CFS assessment tools.

**Symptom domain**	**Recommended tools (adults)**	**Pediatric/special populations**	**Not recommended/limited use**	**Primary application**
Fatigue	CFQ-11; FIS/MFIS; FACIT-F; CIS	PedsQL-MFS	VAS-F (unidimensional); FAI (limited psychometrics)	Clinical grading; research outcomes
Post-exertional malaise	DSQ-PEM; 2-day CPET; FUNCAP (emerging)	—	Generic fatigue tools without PEM items	Diagnosis/phenotyping; monitoring post-exertional change
Cognition	MoCA (screening); TMT; WMS-III	—	MMSE (low sensitivity); CFQ-25 alone (subjective only)	Objective/subjective evaluation; follow-up
Sleep	PSQI; actigraphy; PSG; ESS (for EDS)	—	Sleep diary alone (qualitative only)	Sleep quality profiling; circadian/architecture assessment
Pain	NRS; MPQ; BPI	—	VAS alone (comparability limits)	Pain intensity and impact; trial endpoints
Psychological state	HADS; BDI; (HAMD/HAMA when clinician-rated)	Adolescents: HADS (with caution)	SAS/SDS (education bias); CES-D, GHQ (non-specific)	Screening/monitoring anxiety and depression
Orthostatic intolerance (OI)	HUT (objective); COMPASS-31 (subjective)	—	AST stand-alone (lower specificity)	Autonomic assessment; diagnostic clarification
Multi-dimensional health (QoL/HRQoL)	SF-36; EQ-5D-5L; WHOQOL-BREF	WHOQOL-BREF (time-limited settings)	NHP, SIP (time-consuming, broad)	Global health status; longitudinal tracking

## 12 Conclusion and practical recommendations

This review highlights the heterogeneity of symptom assessment in ME/CFS and underscores the need for standardized, multidimensional, and psychometrically robust instruments. Based on the current evidence, the CFQ-11, DSQ-PEM, PSQI, HADS, and SF-36 emerge as the most validated tools for adult ME/CFS, while newer instruments such as FUNCAP and MBSQ show potential in post-COVID and pediatric populations. Future work should focus on international harmonization, cross-validation, and integration of digital or AI-assisted monitoring systems to support personalized assessment and clinical translation.

Collectively, these efforts will advance the precision, reproducibility, and clinical applicability of ME/CFS symptom assessment, ultimately improving diagnosis and patient outcomes.

## Abbreviations

CFS: chronic fatigue syndrome
CFQ: Chalder fatigue questionnaire
FAI: fatigue assessment instrument
FSS: fatigue severity scale
FIS: fatigue impact scale
CIS: checklist individual strength
VAS-F: visual analog scale for fatigue
pSS: primary Sjögren's syndrome
MFI: multidimensional fatigue inventory
PedsQL-MFS: pediatric quality of life multidimensional fatigue scale
FACIT-F: functional assessment of chronic illness therapy-fatigue
FSMC: fatigue scale for motor and cognitive functioning
DSQ-PEM: DePaul symptom questionnaire–PEM subscale
2-day CPET: two-day cardiopulmonary exercise test
FUNCAP: functional capacity scale
CBT: cognitive behavioral therapy
MoCA: montreal cognitive assessment
CFQ-25: cognitive failures questionnaire
EMQ: everyday memory questionnaire
TMT: trail making test
WMS-III: Wechsler memory scale-III
MMSE: mini-mental state examination
WASI: Wechsler abbreviated scale of intelligence
IQ: intelligence quotient
TP: Toulouse-Piéron test
Stroop: stroop color and word test
PSG: polysomnography
EEG: electroencephalogram
ECG: electrocardiogram
EMG: electromyogram
EOG: electro-oculography
PSQI: Pittsburgh sleep quality index
ESS: Epworth sleepiness scale
EDS: excessive daytime sleepiness
ISI: insomnia severity index
VAS: visual analog scale
NRS: numeric rating scale for pain
MPQ: McGill pain questionnaire
PRI: pain rating index
PPI: present pain intensity
BPI: brief pain inventory
PCS: pain catastrophizing scale
HADS: hospital anxiety and depression scale
HAMA: Hamilton anxiety rating scale
HAMD: Hamilton depression rating scale
SAS: self-rating anxiety scale
SDS: self-rating depression scale
BDI: Beck depression inventory
SCL-90: symptom checklist-90
CES-D: center for epidemiologic studies depression scale
GHQ: general health questionnaire
OI: orthostatic intolerance
NAM: national academy of medicine (US)
POTS: postural tachycardia syndrome
HUT: head-up tilt test
AST: active 10-min standing test
BP: blood pressure
HR: heart rate
COMPASS-31: composite autonomic symptom score-31
OGS: orthostatic grading scale
HRQoL: health-related quality of life
SF-36: 36-item short form health survey
WHOQOL-BREF: abbreviated world health organization quality of life questionnaire
EQ-5D: EuroQol five dimensions questionnaire
MOS-SF: medical outcomes study–short form
RCTs: randomized controlled trials
EQ-VAS: EQ-5D visual analog scale
NHP: Nottingham health profile
SIP: sickness impact profile
MBSQ: Munich-Berlin symptom questionnaire
C19-YRS: COVID-19 Yorkshire rehabilitation scale.
