# Supplementary material for: Assessment of symptoms in myalgic encephalomyelitis/chronic fatigue syndrome: a comparative study of existing scales
Source: Front Neurol. 2025 Nov 18;16:1618272. doi: 10.3389/fneur.2025.1618272 (PMC12668935; doi:10.3389/fneur.2025.1618272)
Supplement: Supplementary file 1 [file Data_Sheet_1.pdf]

Supplementary Table S1. Detailed characteristics and supporting references of fatigue assessment tools in ME/CFS

Scale	Items/domains	Domains	Scoring range	Score–patient relationship	Psychometric validation/ Reliability	Clinical / Research application	Recommended population	Remarks	Supporting References
CFQ	14 / 11	Physical, Mental	0–33	↑ score = ↑ fatigue	Good internal consistency; CFQ-11 shows superior structure	ME/CFS, post-COVID fatigue	Adults	CFQ-11 preferred for improved validity	11-15
FAI	29	Severity, Sensitivity, Psychological impact, Rest/sleep	0–116	↑ score = ↑ fatigue	Limited validation; few ME/CFS-specific studies	ME/CFS, general fatigue	Adults	Distinguishes pathological vs. physiological fatigue	16-17
FSS	9	Fatigue-related impact	9–63	↑ score = ↑ fatigue	Excellent reliability (α>0.9)	ME/CFS, MS, SLE, CRF	Adults	Best for impact on function	18-23
FIS / MFIS	40 / 21	Cognitive, Physical, Psychological	0–160 / 0–84	↑ score = ↑ fatigue	Strong reliability and validity in MS, ME/CFS	ME/CFS, MS	Adults	MFIS more time-efficient	19, 24-27
CIS	20	Fatigue, Attention, Motivati	20–140	↑ score = ↑ fatigue	α≈0.90; high sensitivity and	ME/CFS, cancer, MS	Adults	Focuses on cognitive fatigue component	28-36

		on, Activity			test–retest reliability				
VAS-F	1	Fatigue intensity	0–100 mm	↑ score = ↑ fatigue	Acceptable reliability	ME/CFS, IBD, PSS	Adults	Not diagnostic; unidimensional	27, 37-40
MFI	20	General, Physical, Mental, Motivation, Activity	20–100	↑ score = ↑ fatigue	Validated across multiple chronic diseases	ME/CFS, cancer	Adults	Broad multidimensional structure	41, 45
PedsQL-MFS	18	General, Sleep/rest, Cognitive fatigue	0–100	↓ score = ↑ fatigue	Validated in pediatric and adolescent fatigue	Pediatric ME/CFS	Children	Opposite scoring direction (↓ = worse)	42, 46-47

Supplementary Table S2. Detailed characteristics and supporting references of post-exertional malaise (PEM) assessment tools in ME/CFS

Scale	Items/domains	Domains	Scoring range	Score–patient relationship	Psychometric validation/ Reliability	Clinical / Research application	Recommended population	Remarks	Supporting References
DSQ-PEM	5	frequency, severity, duration of post-exertional symptoms; 6-month recall period	Likert 0–4 for both frequency and severity; PEM considered present when ≥ "moderate" in severity and ≥ "half the time" in frequency	/	Validated across multiple languages and ME/CFS cohorts; internal consistency α > 0.80; applicable in case-definition studies	ME/CFS and post-COVID populations; large-scale epidemiological and diagnostic surveys	Adults / post-COVID patients	Standardized self-report measure; easy administration; recall bias and limited temporal sensitivity	49, 52-56

2-day CPET	2	Two consecutive sessions measuring VO_2max, workload, and anaerobic threshold decline	A ≥8–15% reduction in VO_2max or workload from Day 1 to Day 2 indicates PEM	/	Highly reproducible; objective physiological marker; strong empirical evidence across studies	ME/CFS and long-COVID research; physiological validation and clinical trials	Adults	Objective and reproducible; best-validated physiological measure of PEM; resource-intensive; requires specialized equipment and trained staff	51, 57-61
FUNCAP	3 domains	activity reduction, physical exhaustion, recovery delay	0–10 scale per item; higher scores reflect greater post-exertional functional decline; mean score represents overall impairment	/	Initial development and validation study; promising internal consistency	ME/CFS and post-COVID PEM evaluation; suitable for large-cohort monitoring	Adults	Brief and patient-informed; low burden; limited psychometric validation; diagnostic thresholds not yet established	50

Supplementary Table S3. Detailed characteristics and supporting references of subjective cognitive assessment tools in ME/CFS

Scale	Items/domains	Domains	Scoring range	Score–patient relationship	Psychometric validation/ Reliability	Clinical / Research application	Recommended population	Remarks	Supporting References
MoCA	11	8 domains (attention and concentration, executive function, short-term memory, language, visuoconstruction, abstraction, calculation, orientation)	0–30 (≥26 = normal)	↑ score = ↑ cognitive performance	High sensitivity (90%) and specificity (87%) for mild cognitive impairment; validated across populations	Screening of cognitive dysfunction in ME/CFS, post-COVID, and other neurological disorders	Adults; older adults	Standardized self-report measure; easy administration; recall bias and limited temporal sensitivity	65-69

CFQ-25	25	perceptual, memory, and motor lapses over past 6 months	0–100 (Likert 0–4)	↑ score = ↑ cognitive failures	Good internal consistency (α≈0.89); validated in ME/CFS and psychiatric samples	Applied in ME/CFS, post-COVID, and behavioral studies assessing subjective cognitive complaints	Adults	Objective and reproducible; best-validated physiological measure of PEM; resource-intensive; requires specialized equipment and trained staff	70-74
EMQ	28	5 domains (retrieval, task monitoring, conversation monitoring, spatial memory, active memory)	1–9 per item; higher = greater impairment	↑ score = ↑ cognitive impairment	Validated in community and clinical samples; sensitive to mild memory deficits	Used in ME/CFS and post-COVID to assess subjective memory complaints; comparison with objective tests	Adults	Adults; pediatric adaptation available	75-79

Supplementary Table S4. Detailed characteristics and supporting references of objective cognitive measurement tools in ME/CFS

Scale	Items/domains	Domains	Scoring range	Score–patient relationship	Psychometric validation/ Reliability	Clinical / Research application	Recommended population	Remarks	Supporting References
TMT	2 parts (A/B)	Processing speed, executive function	Completion time (s)	↑ time = ↓ performance	Sensitive to executive dysfunction; reliable; widely used in neuropsychology	Applied in ME/CFS, MS, and other cognitive disorders for processing speed assessment	Adults; suitable for elderly or easily fatigued individuals	Simple and quick; low specificity for sub-processes; single coarse outcome metric	80-83

WMS-III	6 domains, multiple subtests	Verbal comprehension, perceptual organization, processing speed, working memory, auditory and visual memory	Scaled scores (0–20 per subtest)	↑ score = ↑ performance	Strong validity; internal consistency α≈0.85–0.95; cross-cultural adaptations available	Applied in ME/CFS to assess memory and psychomotor speed; standard reference in neuropsychological testing	Adults	Efficient battery for short- and long-term memory; influenced by culture/education and examiner variability	64, 84-86
TP	Several pages, visual scanning task	Attention, perception, processing speed	Dispersion Index (↑=worse), Work Efficiency (↓=worse)	↑ dispersion = ↓ attention stability	Validated in attention and fatigue research; reproducible indices; test–retest reliability >0.80	Used in ME/CFS for evaluating sustained attention and visual processing speed	Adults	Suitable for screening and treatment evaluation of attentional performance; non-diagnostic; influenced by age/education	66, 88-89
Stroop	3 conditions: word reading, color naming,	Processing speed, attention control, response	Completion time and error count	↑ time/errors = ↓ performa	Excellent test–retest reliability; widely	Applied in ME/CFS for evaluating	Adults	Widely applicable; quick to administer; influenced by age,	87, 90-93

	interference	inhibition		nce	used; sensitive to cognitive fatigue	inhibitory control, fatigue-related slowing, and attention deficits		education, vision, sleep; not diagnostic for cognitive impairment

Supplementary Table S5. Detailed characteristics and supporting references of objective sleep measurement tools in ME/CFS

Scale	Items/domains	Domains	Scoring range	Score–patient relationship	Psychometric validation/ Reliability	Clinical / Research application	Recommended population	Remarks	Supporting References
PSG	>10 channels (EEG, ECG, EMG, EOG etc.)	Sleep architecture (TST, latency, efficiency, REM/NREM ratio)	Objective parameters	↑ sleep disturbance = ↓ efficiency	High reproducibility; validated across ME/CFS and insomnia studies	Gold-standard; detailed lab evaluation and therapeutic monitoring	Adults with ME/CFS	Expensive; requires trained technicians; limited accessibility; may disturb natural sleep	99-106
ACT	Wrist-worn motion sensor recording rest–activity cycles	Sleep–wake pattern; circadian rhythm	Device-generated metrics	Movement proxy for sleep quantity and quality	Validated vs. PSG; moderate agreement in ME/CFS and post-COVID populations	Long-term, non-invasive monitoring; home and field research settings	Adults and pediatric patients	Cannot differentiate quiet wakefulness from sleep; influenced by motor disorders; requires sleep diary for accuracy	107-109

Supplementary Table S6. Detailed characteristics and supporting references of subjective sleep measurement tools in ME/CFS

Scale	Items/domains	Domains	Scoring range	Score–patient relationship	Psychometric validation/ Reliability	Clinical / Research application	Recommended population	Remarks	Supporting References
PSQI	19 items; 7 domains (subjective sleep quality, sleep latency, duration, efficiency, disturbances, medication use, daytime dysfunction)	Sleep quality and disturbances over past month	0–21 (Likert 0–3 per item)	↑ score = ↑ poorer sleep quality; ≥5 indicates sleep disturbance	Validated across languages; strong internal consistency; correlated with PSG parameters	Clinical and research evaluation of subjective sleep in ME/CFS	Adults with ME/CFS	Cannot determine specific causes of sleep disturbance; limited for long-term evaluation	110-116
ESS	8 items assessing daytime sleepiness in common daily situations	Daytime sleepiness and alertness	0–24 (Likert 0–3 per item)	↑ score = ↑ daytime sleepiness (0–9 normal; 10–15 mild; ≥16 seve	Good reliability; widely validated; applicable in ME/CFS studies	Screening of excessive daytime sleepiness and fatigue-related disorders	Adults with ME/CFS	Not diagnostic for sleep disorders; evaluates daytime sleepiness only	117-119

				re)				
ISI	7 items assessing insomnia type, severity, and daily impact over past month	Insomnia severity and impact	0–28 (Likert 0–4 per item)	↑ score = ↑ insomnia severity (0–7 none; 8–14 mild; 15–21 moderate; 22–28 severe)	High reliability; validated in insomnia and ME/CFS-related populations	Screening and outcome tracking in insomnia and ME/CFS studies	Adults with ME/CFS	Emotionally influenced; limited ME/CFS-specific validation

Sleep Diary	Daily record of nighttime sleep and daytime performance	Subjective sleep–wake pattern and behavior	N/A (qualitative daily entries)	↑ subjective accuracy vs. retrospective recall	Moderate reliability; complements actigraphy; widely used in behavioral sleep studies	Adjunct tool for sleep–wake monitoring and treatment evaluation	Adults with ME/CFS	Highly subjective; low reliability; auxiliary to objective tools

Supplementary Table S7. Detailed characteristics and supporting references of pain measurement tools in ME/CFS

Scale	Items/domains	Domains	Scoring range	Score–patient relationship	Psychometric validation/ Reliability	Clinical / Research application	Recommended population	Remarks	Supporting References
VAS	Single item assessing current pain intensity using a 100-mm line anchored by "no pain" and "severe pain"	Pain intensity (unidimensional)	0–100 mm	↑ score = ↑ pain intensity	High sensitivity and test–retest reliability; validated across clinical pain populations including ME/CFS	Clinical pain evaluation and treatment effect comparison	Adults with ME/CFS	Requires abstract reasoning; unidimensional; not comparable across individuals	128, 131-134
NRS	Single item rating pain on an 11-point scale (0 = no pain, 10 = worst pain)	Pain intensity (unidimensional)	0–10	↑ score = ↑ pain intensity	High concordance with VAS; widely validated and reproducible	trials and patient-reported pain monitoring	Adults with ME/CFS	Requires numerical understanding; influenced by language and cognition; lower sensitivity than VAS	129, 135-138

MPQ	78 descriptors across 20 subclasses covering sensory, affective, and evaluative pain dimensions	Multidimensional pain profile	N/A (PRI, NWC, PPI indices)	↑ score = ↑ pain perception/severity	Strong psychometric foundation; sensitive to treatment effects; validated in chronic pain and ME/CFS studies	Differentiation of nociceptive vs. neuropathic pain; comprehensive pain characterization	Adults with ME/CFS	Lengthy; literacy-dependent; time-consuming; influenced by gender and ethnicity	130, 139-142
BPI	11 items assessing pain intensity and interference across seven domains (activity, mood, sleep, enjoyment, etc.)	Pain intensity and functional interference	0–10 (Likert scale per item)	↑ score = ↑ pain or interference	Validated across multiple chronic pain conditions; good internal consistency; limited ME/CFS-specific data	Multidimensional pain assessment in clinical and epidemiological research	Adults with ME/CFS	Cannot diagnose neuropathic pain; limited ME/CFS validation	143, 145-147
PCS	13 items assessing pain catastrophizing across rumination, magnification, and helplessness dimensions	Pain-related cognition and affect	0–52 (Likert 0–4 per item)	↑ score = ↑ catastrophizing	Strong psychometric validity; validated across chronic pain and ME/CFS populations	Assessment of pain-related maladaptive cognition and coping strategies	Adults with ME/CFS	Indirect measure of pain; subjective; prone to response bias	144, 148-149

Supplementary Table S8. Detailed characteristics and supporting references of psychological status assessment tools in ME/CFS

Scale	Items/domains	Domains	Scoring range	Score–patient relationship	Psychometric validation/ Reliability	Clinical / Research application	Recommended population	Remarks	Supporting References
HADS	14 items (7 anxiety + 7 depression); 2 subscales: HADS-A, HADS-D	Anxiety and depression	0–42 (Likert 0–3 per item)	↑ score = ↑ symptom severity; ≥9 indicates anxiety or depression	Good internal consistency (α≈0.80–0.90); validated in ME/CFS and general medical settings	Screening and evaluation of anxiety/ depression comorbidity in ME/CFS	Adults and adolescents with ME/CFS	Limited ME/CFS-specific validation; difficulty separating anxiety from depression; requires trained raters	151-154
HAMA	14 items assessing psychological and somatic anxiety symptoms	Anxiety (psychological and somatic)	0–56 (Likert 0–4 per item)	↑ score = ↑ anxiety severity; ≥8 indicates clinical anxiety	Reliable and valid; sensitive to treatment response; widely used in psychiatric and ME/CFS studies	Evaluation of anxiety symptom severity and treatment response	Adults with ME/CFS	Overlap with depression items; requires professional administration	155-163

HAMD	17 items across 5 domains (anxiety, somatic symptoms, depression, insight, sleep)	Depression (psychological and somatic)	0–52 (Likert 0–4 per item)	↑ score = ↑ depression severity; ≥8 suggests depressive disorder	High reliability; validated in psychiatric and ME/CFS research; sensitive to treatment effects	Clinical and research evaluation of depressive symptoms in ME/CFS	Adults with ME/CFS	Overlap with HAMA; time-consuming; may not separate anxiety from depression clearly	
SAS	20 items (15 negative, 5 positive) assessing anxiety symptoms	Affective and somatic anxiety symptoms	20–80 (raw) / 25–100 (standardized)	↑ score = ↑ anxiety severity; ≥50 suggests anxiety disorder	Good internal consistency (α≈0.85); validated in ME/CFS and psychiatric cohorts	Screening and monitoring of anxiety symptoms and treatment response	Adults and clinical populations with ME/CFS	Self-reported; may overestimate comorbidity with depression; limited literacy sensitivity	164-169
SDS	20 items (10 negative, 10 positive) assessing emotional and somatic depression	Depressive symptoms (emotional + somatic)	20–80 (raw) / 25–100 (standardized)	↑score = ↑depression severity; ≥53 indicates depression	High reliability (α≈0.88); validated in ME/CFS and fatigue-related studies	Screening of depressive symptoms and treatment effect tracking	Adults with ME/CFS	Non-diagnostic; limited accuracy in low-education populations; may overlap with fatigue scales	

BDI	21 items assessing cognitive, affective, and somatic symptoms	Depression severity (cognitive–affective focus)	0–63 (Likert 0–3 per item)	↑score = ↑depressive symptom severity (0–13 minimal; 14–19 mild; 20–28 moderate; ≥29 severe)	Highly reliable (α≈0.90); validated across psychiatric and ME/CFS samples	Screening and clinical evaluation of depressive symptoms	Adults with ME/CFS	Does not differentiate depression subtypes; not suitable for low-literacy groups	170-171
CES-D	20 items covering depressive mood, somatic, and interpersonal symptoms	Depression and mood disturbance	0–60 (Likert 0–3 per item)	↑score = ↑depressive symptoms; ≥16 indicates depression	High sensitivity and validity; widely applied in epidemiologic and ME/CFS research	Screening depressive symptoms in population or cohort studies	Adults with ME/CFS	Cannot diagnose depression; may be affected by self-report bias	172-173

SCL-90	90 items assessing 9 psychological dimensions including depression, anxiety, hostility, phobia, psychoticism	Comprehensive psychological symptom profile	0–4 per item; T-score norms (≥63 clinically significant)	↑score = ↑psychological distress	Strong internal consistency (α≈0.97); validated in ME/CFS, chronic illness, and psychiatric populations	Broad screening of psychological distress and comorbid symptoms in ME/CFS	Adults with ME/CFS	Highly subjective; not ME/CFS-specific; limited diagnostic precision	174-175
GHQ	12–60 items depending on version; measures somatic, anxiety, social dysfunction, depression domains	General psychological health screening	0–36 (GHQ-12), 0–60 (GHQ-30)	↑score = poorer psychological well-being; ≥12 indicates distress	High reliability and sensitivity; validated across clinical and general populations	Population screening and mental health monitoring in ME/CFS cohorts	Adults with ME/CFS	Designed for general mental health screening; less suitable for ME/CFS-specific symptom tracking	176-177

Supplementary Table S9. Detailed characteristics and supporting references of orthostatic intolerance (OI) objective measurement tools in ME/CFS

Scale	Items/domains	Domains	Scoring range	Score–patient relationship	Psychometric validation/ Reliability	Clinical / Research application	Recommended population	Remarks	Supporting References
HUT	Supine and tilted (70°) positions; BP and HR measured continuously	Autonomic function; hemodynamic response to upright posture	BP, HR, cerebral blood flow changes; abnormal if HR↑ ≥30 bpm or BP↓ ≥20/10 mmHg within 10 min	↑ HR or ↓ BP = ↑OI severity	Validated across ME/CFS and autonomic dysfunction studies; high reproducibility; consistent diagnostic utility	Diagnosis of orthostatic hypotension, POTS, syncope; evaluation of autonomic dysfunction in ME/CFS	Adults with ME/CFS and orthostatic intolerance symptoms	Requires professional supervision; risk of syncope; possible false-positive responses; time-consuming	181-185
AST	10-min upright standing without movement; BP and HR continuously recorded	Sympathetic response and cardiovascular adaptation to posture change	HR and BP measured every minute; abnormal if HR↑ ≥30 bpm or BP↓ ≥20/10 mmHg	↑ HR = ↑ orthostatic intolerance severity	Good internal reliability; moderately correlated with HUT findings; limited ME/CFS-specific validation	Screening for OI and autonomic imbalance; assessment of postural tachycardia in ME/CFS	Adults and clinical trial participants with suspected OI	Lower specificity and sensitivity than HUT; prone to motion artifacts and interpretation bias	186-189

Supplementary Table S10. Detailed characteristics and supporting references of subjective orthostatic intolerance (OI) assessment tools in ME/CFS

Scale	Items/domains	Domains	Scoring range	Score–patient relationship	Psychometric validation/ Reliability	Clinical / Research application	Recommended population	Remarks	Supporting References
COMPASS-31	31 items across 6 domains: orthostatic intolerance, vasomotor, secretomotor, pupillomotor, gastrointestinal, and bladder function	Autonomic symptom severity; orthostatic intolerance	0–100 total score (weighted per domain)	↑ score = ↑ autonomic symptom severity	Good internal consistency (α≈0.80); validated across autonomic disorders and fibromyalgia; limited ME/CFS-specific data	Assessment of autonomic dysfunction and OI severity; monitoring symptom changes and treatment response	Adults with ME/CFS or suspected autonomic dysfunction	Partly subjective; not disease-specific; limited ME/CFS validation; may miss subclinical OI	190-193
OGS	5 items assessing frequency, severity, triggering scenarios, duration of standing, and impact on daily	Orthostatic intolerance and postural symptoms	0–20 (Likert 0–4 per item)	↑ score = ↑ severity of orthostatic symptoms	Moderate internal reliability; correlated with autonomic function tests; limited ME/CFS-specific psychometric validation	Screening and evaluation of OI symptoms in ME/CFS and autonomic disorders	Adults with ME/CFS and orthostatic intolerance symptoms	Useful for preliminary screening; not diagnostic; lower specificity compared with physiological tests	194-197

life

Supplementary Table S11. Detailed characteristics and supporting references of multidimensional health and quality of life（QoL） assessment tools in ME/CFS

Scale	Items/domains	Domains	Scoring range	Score–patient relationship	Psychometric validation/ Reliability	Clinical / Research application	Recommended population	Remarks	Supporting References
SF-36	36 items across 8 domains: physical functioning, role-physical, bodily pain, general health, vitality, social functioning, role-emotional, mental health	Physical, psychological, and social functioning	0–100 (higher = better HRQoL)	↑ score = ↑ HRQoL	High internal consistency (α≈0.85–0.95); widely validated; applied in >30% of ME/CFS RCTs	Assessment of HRQoL and functional impairment; outcome evaluation in clinical trials	Adults with ME/CFS and chronic fatigue disorders	May not distinguish moderate-to-severe cases; lengthy for large-scale surveys	199-203
EQ-5D	5 dimensions: mobility, self-care, usual activities, pain/discomfort, anxiety/depression; includes EQ-VAS (0–100 scale)	Physical and mental health; self-perceived health status	EQ-5D index: −0.59–1.00; EQ-VAS: 0–100	↑ score = ↑ health status; 0=worst, 1=best	Validated across > 100 countries; EQ-5D-5L reduces ceiling effect; reliable in ME/CFS and chronic illness cohorts	QoL evaluation, health utility estimation, and intervention outcome analysis in ME/CFS	Adults and clinical research participants	EQ-5D-3L has low sensitivity to small health changes; 5L version more accurate but complex	27, 204-210

WHOQOL-BREF	26 items across 4 domains: physical health, psychological health, social relationships, environment	Global and domain-specific HRQoL	0–100 (transformed domain scores)	↑ score = ↑ health-related quality of life	Good reliability (α≈0.70–0.90); validated across 20+ languages; cross-cultural applicability; used in ME/CFS studies	Assessment of general and HRQoL; suitable for time-limited studies	Adults with ME/CFS and family caregivers	Does not capture 24 detailed facets of WHOQOL-100; may omit ME/CFS-specific concerns	211-213
NHP	Part 1: 38 items in 6 domains (energy, pain, emotional reactions, sleep, social isolation, physical mobility); Part 2: 7 items on daily life issues	Physical, psychological, and social health aspects	0–100 weighted domain scores (higher = worse health)	↑score = ↓HRQoL	Validated in chronic illness; good sensitivity to sleep and pain changes; limited ME/CFS-specific evidence	Assessment of HRQoL impairment in ME/CFS and related disorders	Adults with ME/CFS, fibromyalgia, or chronic illness	First part lacks total score; second part less specific for ME/CFS; vague item weighting	214-215

SIP	136 items assessing 12 dimensions: sleep, eating, work, home management, recreation, ambulation, mobility, body care, social interaction, alertness, communication, emotional behavior	Functional impairment and HRQoL limitations	0–100 (percentage of dysfunction; higher = worse HRQoL)	↑score = ↓HRQoL / ↑impairment	Excellent internal consistency (α≈0.94–0.98); comprehensive coverage; validated in multiple chronic diseases	Comprehensive HRQoL and disability evaluation; health economics and intervention impact studies	Adults with ME/CFS and severe chronic fatigue	Lengthy; requires patient communication; may overestimate impairment unrelated to fatigue	216-217
